# Current Data Gaps in Modeling Essential Worker Absenteeism Due to COVID-19

**DOI:** 10.1017/dmp.2020.353

**Published:** 2020-09-10

**Authors:** Zackery White, Jeff Schlegelmilch, Jackie Ratner, Gunjan Saxena, Kevin Wongsodirdjo, Susanna Aguilar, Daniel Kushner, Jim Ortega, Aleksi Paaso, Shay Bahramirad

**Affiliations:** The National Center for Disaster Preparedness at The Earth Institute, Columbia University, New York, NY; Commonwealth Edison, Chicago, IL

**Keywords:** absenteeism, COVID-19, critical Infrastructure, modelling, pandemics

## Abstract

With the uncertain physical and mental health implications of COVID-19 infection, companies have taken a myriad of actions that aim to reduce the risk of employees contracting the virus, with most grounded in reducing or eliminating in-person interactions. Our preliminary analysis indicates that while there is some data to support modelling absenteeism, there are gaps in the available evidence, requiring the use of assumptions that limit precision and efficacy for decision support. Improved data on time-to-recovery after hospitalization, absenteeism due to family or other household member illness, and mental health’s impact on returning to work will support the development of more robust absenteeism models and analytical approaches.

With the uncertain physical and mental health implications of the coronavirus disease (COVID-19) infection, companies have taken a myriad of actions that aim to reduce the risk of employees contracting the virus, with most grounded in reducing or eliminating in-person interactions. However, utilities and other essential industries may be unable to fully eliminate the need for in-person attendance. The National Center for Disaster Preparedness (NCDP) at Columbia University’s Earth Institute has been supporting the electric utility company Commonwealth Edison (ComEd) to help identify parameters for modeling workforce absenteeism. As part of this effort, NCDP has developed a research dashboard to track publications that are non-, pre-, and post- peer review to support analytical parameter assumptions. While more than 80 publications have been analyzed, there are many data gaps that pose a challenge for the accurate forecasting of employee absenteeism (Figure 1).

**FIGURE 1:**
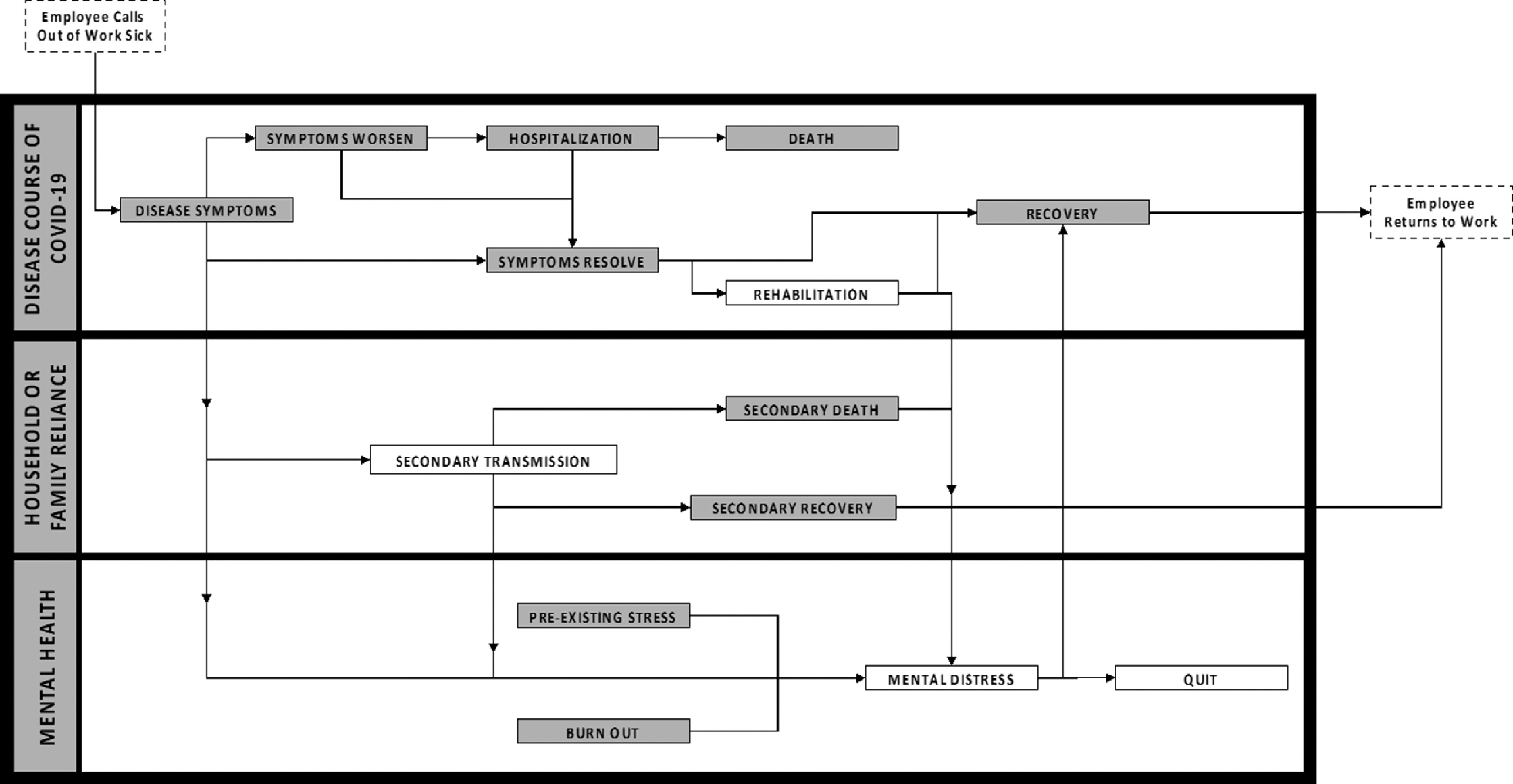
Swim Lane Diagram of the Conceptual Inner Workings of Employee Absenteeism and Events That Alter the Timeline for the Return to Work. (The shaded boxes indicate parameter values that have evidence to support the development of more robust absenteeism models. This diagram does not represent all events that an individual may experience.)

## DISEASE COURSE OF COVID-19

In the simplest scenario, employees who contract COVID-19 would miss work and return 24 hours after their fever resolves and their other symptoms improve, and at least 10 days have passed since symptom onset.^1^ However, the disease course is not well studied in moderate cases, as most studies use hospital records, which reflect severe cases and do not capture the time frame post-discharge until returning to work. Two pre-peer-reviewed studies with longitudinal follow-up for non-hospitalized patients revealed that, on average, symptoms in outpatients resolved after 3 weeks with a standard deviation of 1.25 weeks, not accounting for the possibility that an employee’s symptoms could worsen, requiring hospitalization and extending his or her absence.^2,3^ We have found no data nor proxy for estimating the recovery time after discharge, though we would expect that those hospitalized, especially those requiring intensive care, would need extensive time to recuperate prior to re-entering the workforce.

## OTHER IMPACTS ON ABSENTEEISM

Absenteeism due to an exposure requiring quarantine, and/or in order to care for a family or other household member, also has an unspecified impact on absenteeism rates. This is potentially exacerbated by an overburdened testing system and many households being unable to accommodate at-home self-isolation in the United States.^4^ In our review, we did not find any data on absenteeism related to illness of a family or other household member. Additionally, the prolonged nature of the pandemic has taken an emotional toll: 53% of US adults say that COVID-19-related stress has had a negative impact on their mental health, up from 39% in May.^5^ This could potentially result in increased absenteeism or an extended or permanent leave of absence.

Our preliminary analysis indicates that, while there are some data to support modeling absenteeism, there are gaps in the available evidence, requiring the use of assumptions that limit precision and efficacy for decision support. Improved data on time-to-recovery after hospitalization, absenteeism due to family or other household member illness, and mental health’s impact on returning to work will support the development of more robust absenteeism models and analytical approaches. We also acknowledge that this analysis and approach are not exhaustive, and that additional factors for absenteeism should be identified and analyzed as the evidence-base is further developed.

## References

[1] CDC. Discontinuation of isolation for persons with COVID-19 not in healthcare settings. United States Centers for Disease Control and Prevention. Updated July 20, 2020 https://www.cdc.gov/coronavirus/2019-ncov/hcp/disposition-in-home-patients.html. Accessed August 19, 2020.

[2] O’Keefe JB , Tong EJ , Datoo O’Keefe GA , Tong DC. Predictors of disease duration and symptom course of outpatients with acute COVID-19: a retrospective cohort study. medRxiv. 2020.

[3] Mizrahi B , Shilo S , Rossman H , et al. Longitudinal symptom dynamics of COVID-19 infection in primary care. medRxiv. 2020. 10.1038/s41467-020-20053-y PMC7718370 33277494

[4] Sehgal AR , Himmelstein DU , Woolhandler S. Feasibility of separate rooms for home isolation and quarantine for COVID-19 in the United States. Ann Intern Med. 2020. 10.7326/M20-4331 PMC7392146 32692931

[5] KFF.org. Hamel L , Kearney A , Kirzinger A , et al. *KFF Health Tracking Poll – July 2020.* Henry Kaiser Family Foundation. July 27, 2020 https://www.kff.org/coronavirus-covid-19/report/kff-health-tracking-poll-july-2020/. Accessed September 1, 2020.

